# An Overview of Social Media Use in the Field of Public Health Nutrition: Benefits, Scope, Limitations, and a Latin American Experience

**DOI:** 10.5888/pcd17.200047

**Published:** 2020-08-06

**Authors:** Kenny Mendoza-Herrera, Isabel Valero-Morales, Maria E. Ocampo-Granados, Hortensia Reyes-Morales, Fernanda Arce-Amaré, Simón Barquera

**Affiliations:** 1 Center for Nutrition and Health Research, National Institute of Public Health, Cuernavaca, Morelos, México; 2 National Institute of Public Health, Cuernavaca, Morelos, México; 3 Center for Information on Public Health Decisions, National Institute of Public Health, Cuernavaca, Morelos, México; 4 International Society of Nephrology, Brussels, Belgium

## Abstract

Social media platforms are low-cost tools that can be used to address issues in public health nutrition, especially in countries where health-related institutions experience economic limitations. We aimed to emphasize the benefits of using social media to promote health that have been documented to date. To show social media’s positive impact on population health literacy, we briefly describe an inexpensive systematic communication strategy implemented in our research center through 2 social media platforms, the lessons learned, and the strategy’s short-term results. Because social media use in public health is a new field of study, this perspective also focuses on the current limitations and gaps in evidence that need to be addressed to translate the best practices into policy recommendations. In conclusion, the perspective highlights the role that health actors and governments should take to maximize the benefits of social media use.

Social media online platforms, such as Facebook and Twitter, are promising instruments to improve population health. In 2012, the World Health Organization (WHO) launched the global eHealth strategy to encourage the promotion, development, and evaluation of actions that involve these platforms (1,2). Social media can encourage citizen participation, optimize health systems, be an interactive space for science dissemination, support health policies, and promote healthy behaviors. This perspective emphasizes the benefits and limitations of social media, considering that they could effectively address public health nutrition problems.

Interventions involving social media can influence behaviors to improve lifestyles and metabolic indicators of noncommunicable diseases (NCDs) (3–5). In an effort to prevent NCDs, social media interventions can help people increase their physical activity levels (6) and reduce their sugar and fat consumption (7,8), enhance motivation among online health programs users (3,5,9), and deliver nutritional or diabetes education (4,7). Social media is also beneficial outside controlled interventions because it can increase citizens’ awareness of public issues and allow them to take a more active and better informed role in their communities (10–13). Twitter content analyses show a significant participation in discussions related to childhood obesity or strategies against alcohol overconsumption (14,15).

Social media platforms can strengthen health professionals’ counseling (16), empower patients to learn about their conditions (17), and promote equity in health care services. Social media interventions can be successful in vulnerable populations, including low-income sectors, rural areas, and minority ethnic groups (18).

The use of social media has gained recognition among scholars (19–21), because its use is associated with higher article citation (22–24) and increased accessibility of scientific evidence to the general public (25–28). Considering these contributions, social media platforms are powerful instruments for health education (29–31) that diverse age groups can use daily to learn and share knowledge (32).

Social media platforms are relevant information sources for policy makers (33–35). Because these communication channels are continually updated, they provide current indicators for health policy analysis and action. Text, photos, videos, locations, and social networks can be used for public health surveillance, optimizing policy interventions, geographically identifying vulnerable groups in need of resource allocation, and designing policies that consider how individuals interact inside communities (36).

Social media reaches millions of users on free access platforms (37). Two-thirds of adults from 40 countries are internet users, of which 76% use social media platforms (38). Thus, strategic health promotion through these tools can optimize resources commonly allocated to expensive campaigns in conventional mass media. This optimization could be especially beneficial in nations such as Mexico, where the health budget diminished in the last decade (39,40) and the average percentage of the Gross Domestic Product allocated to health is 35.6% lower than in member countries of the Organization for Economic Co-operation and Development (41).

Agencies such as WHO, the World Bank, and the United Nations Children’s Fund regularly use social media for health promotion and have reached millions of followers worldwide, given that they have accounts adapted to regional contexts, cultural backgrounds, languages, and local issues (Table). Number of publications and their interactions per 1,000 users are metrics that provide insight about users’ engagement level and allow for comparisons between accounts (42). Higher engagement is associated with a larger number of publications.

**Table:** Selected Facebook and Twitter Health and Nutrition-Related Accounts by Sector

Organization, Facebook/Twitter Handle	Facebook Page		Twitter Account		No. of Posts and Engagements in a 5-Week Period on Facebook Pages^c^	
	No. of Likes^a^ (Thousands)	Year Created^a^	No. of Followers^a^ (Thousands)	Year Created^a^	Average Posts Per Week	Minimum and Maximum Interactions Per 1,000 Fans^d^
**International agencies**						
UNICEF, @unicef^b^	7,829.9	2009	7,800.0	2009	NA	NA
WHO, @WHO^b^	4,441.6	2010	5,000.0	2008	NA	NA
World Bank, @WorldBank^b^	2,593.5	2010	3,100.0	2009	NA	NA
**Government agencies**						
Canadian Food Inspection Agency, @CFIACanada/@InspectionCan	51.6	2013	56.4	2009	22.2	15.6–68.6
CDC (US), @CDC/@cdcgov	912.8	2009	1,200.0	2010	20.2	13.1–77.6
Chilean Agency for Food Safety and Quality (Chile), @achipia.oficial/@ACHIPIA	8.2	2014	5.3	2011	1.8	0–9.6
CINyS (Mexico), @CINyS.INSP/@1CINyS	38.8	2017	6.5	2017	11.6	26.6–83.1
Mexican National Institute of Public Health, @insp.mx/@inspmx	82.9	2011	34.7	2010	20.6	40.6–65.2
Model Market (Uruguay), @mercadomodelouruguay/MercadoModeloUy	22.4	2012	1.8	2016	5.6	3.4–15.5
National Health Surveillance Agency (Brazil), @AnvisaOficial/@anvisa_oficial	91.8	2017	56.6	2009	23.4	27.0–74.6
The Institute of Nutrition of Central America and Panama, @incap.int/@INCAP_NUTRICION	22.3	2012	1.4	2013	3.6	7.0–23.5
The Choose Healthy Living System (Chile), @EligeVivirSano^b^	534.5	2011	66.2	2011	4.2	0.1–0.6
**Ministries of Health**						
Brazil, @minsaude^b^	2,167.1	2010	719.7	2009	25.0	14.3–36.2
Canada,@HealthyCdns, @GovCanHealth	115.2	2009	235.7	2009	21.6	6.8–41.9
Chile, @ministeriosaludchile/@ministeriosalud	309.1	2011	247.5	2010	65.0	5.2–8.5
Mexico, @SecretariadeSaludMX/@SSalud_mx	844.2	2011	589.5	2010	105.0	24.8–158.7
Uruguay, @MSPUruguay^b^	39.6	2015	19.1	2015	3.6	0–84.1
United States of America, @HHS, @hhsgov	228.4	2013	788.7	2009	10.6	2.2–8.7
**Civil society and nonprofit organizations**						
Brazilian Institute of Consumer Protection, @idecbr/@idec	250.6	2010	41.2	2009	6.6	1.6–5.9
Canadian Public Health Association, @cpha.acsp/ @CPHA_ACSP	3.9	2009	6.6	2011	5.4	0.8–8.3
Five a Day in Chile, @5aldia.cl/@5aldiachile	155.7	2012	11.2	2010	7.6	1.2–15.8
Non-communicable Diseases Alliance (USA), @ncdalliance^b^	3.1	2015	20.5	2011	8.4	15.0–236.2
Observatory of the Health System of Uruguay, @OSalud	No account	NA	0.7	2012	NA	NA
The Power of the Consumer (Mexico), @elpoderdelc^b^	458.3	2010	37.0	2010	25.0	12.0–43.5

Abbreviations: CDC, Centers for Disease Control and Prevention; CINyS, Center for Nutrition and Health Research; NA, not applicable; WHO, World Health Organization.

a Data were taken from Facebook and Twitter. Accessed January 14, 2020.

b Names of Facebook page and Twitter account are the same.

c Data extracted from SocialBakers (socialbakers.com), in the period February 6, 2020, to March 12, 2020.

d Defined as the sum of likes, commentaries, and shares divided by the number of fans the page has on the day of the post and multiplied by 1,000.

Government agencies, Ministries of Health, and civil societies in the Americas promote health and nutrition through social media. For example, the Canadian Food Inspection Agency and a Chilean nutritional program have tweeted a considerable amount of information on health strategies. The Brazilian Ministry of Health supports health campaigns, and American civil society groups promote policies against NCDs. Mexican institutions, such as the Mexican National Institute of Public Health (INSP), adopted social media to interact directly with users and promote better health (Table).

In 2017, the Center for Nutrition and Health Research (CINyS), a division of the INSP, started a systematic social media communication strategy focused on dissemination of science and nutrition policies aimed at addressing obesity, including regulations for food advertising, taxes, and front-of-package labeling (FOPL) systems. It also considers health-related international days, issues relevant to the national health agenda, and diffusion of academic events organized by the center (strategy’s content at https://bit.ly/2UgePkd; Facebook: @CINyS.INSP; Twitter: @1CINyS). A team of 5 nutrition researchers, 2 graduate students, a graphic designer, and a community manager develop evidence-based visual content, which mainly includes original infographics with title, introduction, key messages, recommendations, and information sources. A monthly plan is developed to schedule the dissemination of these elements in 6 or more posts per week.

Despite the lack of paid advertising, the number of the center’s fans has consistently grown. CINyS has 38,800 likes on Facebook with a monthly average increase of 2,029 from September 2019 to February 2020. On Facebook, for example, the monthly mean number of engagements for total publications in this 6-month period was 46,822 (likes, shares, or commentaries). CINyS’s infographics were shared by relevant health-related accounts, which sparked conversation and generated synergy. Examples can be accessed at https://bit.ly/2xMAHMw and at https://bit.ly/2vy5yf4. These examples show that users interact more with visuals inspired by popular culture with elements such as fun memes or cartoons than they do with content lacking these visual elements. Diffusion of our academic events on social media has generated positive results. For instance, CINyS organized a conference to celebrate the first unified World Obesity Day, which was widely promoted on social media (invitation at https://bit.ly/3blIz65). More than 1,200 participants gathered for this event, and it was live-streamed, reaching more than 39,000 users (video at https://bit.ly/3bmyea8). Such high attendance levels had not been seen before this strategy. Although we do not have enough elements to compare our strategy with other campaigns in detail, we consider that it has been low-cost. Only the graphic designer and community manager work full time, with minor activities performed by junior investigators and students who are covered by soft funding and scholarships.

CINyS has achieved relevant participation in health policy discussions through its strategy. For example, the process to adopt a new FOPL system in Mexico was a topic on Twitter in which several key actors posted. We developed 12 infographics and 114 tweets between October 2019 and March 2020 to support it. These materials were shared by other health accounts, and this activity reached 387,600 impressions with an engagement of 20,300 (Figure). The Mexican government reported an unprecedent number of comments on the new FOPL regulatory framework from citizens and different actors.

**Figure:**
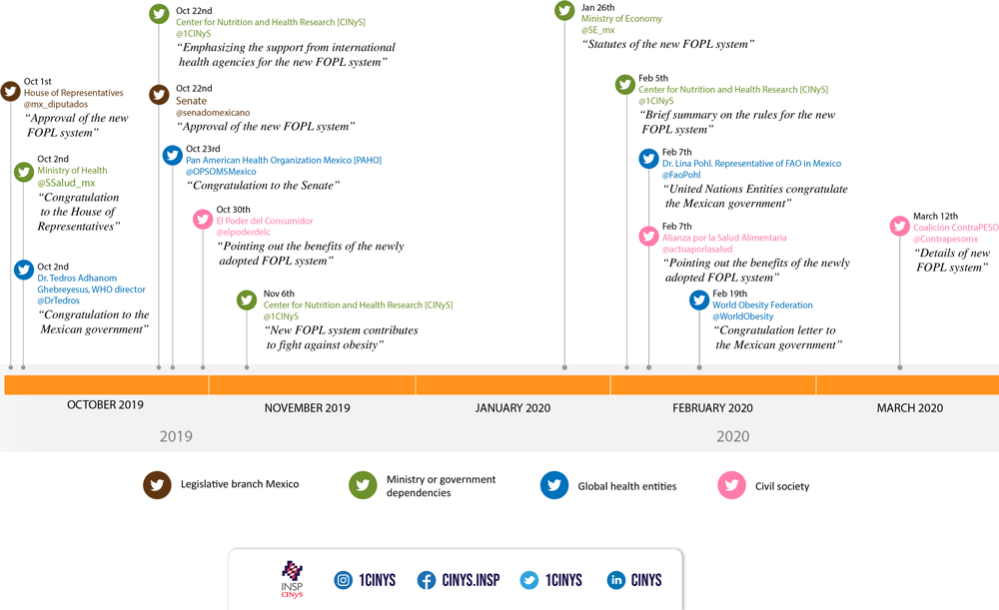
Timeline with selected tweets related to a discussion on the adoption of the new FOPL system in Mexico, October 2019 to March 2020. El Poder del Consumidor is a civil society that works for consumers’ rights, Alianza por la Salud Alimentaria is a group of organizations that work on actions against obesity and undernutrition, and Coalición ContraPESO is a civil society formed by 40 organizations that works on actions against obesity. Abbreviations: CINyS, Center for Nutrition and Health Research; FOPL, front-of-package labeling; WHO, World Health Organization.

This experience highlights the potential of social media to be used for health promotion, even with limited resources. CINyS looks to expand its strategy through paid advertising, content translation into other languages, development of other types of multimedia materials, and an expansion to new social media platforms to reach younger audiences. We are not certain whether the strategy’s growth will continue to be linear or if it will reach a saturation point in terms of the number of interested users.

Year of creation modifies how health accounts reach users. Accounts created first have higher levels of interaction metrics than newer ones, reflecting the importance of early social media adoption. In contrast, most big food companies, which use social media to advertise their products and have created accounts more recently, have attained greater popularity partly due to heavily funded marketing. For example, Coca-Cola’s Facebook page, opened in 2014, has 107 million likes. This marketing is difficult to counteract, considering that health institutions do not sufficiently promote healthy practices (43) and governments have not allocated equivalent resources for health promotion on social media.

Another challenge for health promotion through social media is misleading information (44), which is commonly supported by false accounts (45). However, participation from concerned users and trustworthy institutions helps to overcome this shortcoming, which is only possible in an interactive platform and not with one-directional media. To reinforce this positive response, regulatory entities and academia must work together to certify digital profiles and provide reliable accounts lists.

The legal framework for eHealth in the Americas must be enhanced to extend the social media benefits. WHO has reported that no national policy exists that makes specific reference to social media use in health programs and services or other public health actions in Mexico (46). In contrast to what occurs for other health topics in the national agenda, such as vaccination or obesity prevention, the government has not created an official commission to lead the development of eHealth policies aimed at regulating social media use.

Notwithstanding, the documented benefits and the popularity of social media, its causal impacts on health (18), and the mechanisms through which its content influences policy makers’ practice (47,48) remain unclear. This lack of evidence is partly explained by the lack of rigor in current studies that aim to assess this phenomenon (6). Since the effects of social media on health have been explored mainly by using platforms created for study purposes (18), the knowledge on the potential of Facebook, Twitter, or YouTube should be explored further. Development of high-quality methods to evaluate the impact of both commercial and noncommercial platforms on health outcomes is essential to translate best practices into recommendations. In an effort to extend social media benefits, all health entities in the Americas should adopt them to complement their communication activities. Governments should form commissions of experts to improve digital regulations that focus on preventing misleading information through social media platforms and officially specify the correct use of those platforms in differing health domains.
